# Pituitary-adrenocortical adjustments to transport stress in horses with previous different handling and transport conditions

**DOI:** 10.14202/vetworld.2016.856-861

**Published:** 2016-08-14

**Authors:** E. Fazio, P. Medica, C. Cravana, and A. Ferlazzo

**Affiliations:** Department of Veterinary Sciences, University of Messina, Polo Universitario Annunziata, 98168 Messina, Italy

**Keywords:** adrenocorticotropic hormone, cortisol, horse, previous transport experience, social context, transport stress

## Abstract

**Aim:**

The changes of the hypothalamic pituitary adrenal (HPA) axis response to a long distance transportation results in increase of adrenocorticotropic hormone (ACTH) and cortisol levels. The purpose of the study was to quantify the level of short-term road transport stress on circulating ACTH and cortisol concentrations, related to the effect of previous handling and transport experience of horses.

**Materials and Methods:**

The study was performed on 56 healthy horses after short-term road transport of 30 km. The horses were divided into four groups, Groups A, B, C, and D, with respect to the handling quality: Good (Groups A and B), bad (Group D), and minimal handling (Group C) conditions. According to the previous transport, experience horses were divided as follows: Horses of Groups A and D had been experienced long-distance transportation before; horses of Groups B and C had been limited experience of transportation.

**Results:**

One-way RM-ANOVA showed significant effects of transport on ACTH changes in Groups B and C and on cortisol changes in both Groups A and B. Groups A and B showed lower baseline ACTH and cortisol values than Groups C and D; Groups A and B showed lower post-transport ACTH values than Groups C and D. Groups A, B, and C showed lower post-transport cortisol values than Group D. Only Groups A and B horses have shown an adequate capacity of stress response to transportation.

**Conclusion:**

The previous transport experience and quality of handling could influence the HPA axis physiological responses of horses after short-term road transport.

## Introduction

The hypothalamic pituitary adrenal (HPA) axis is a major provider to the homeostatic mechanisms involved in the response to stress. The measurement of the different hormones that are part of the HPA, like adrenocorticotropic hormone (ACTH) and cortisol, is considered the standard approach to evaluate animal stress and welfare [1,2]. Baseline circulating ACTH and cortisol values may be useful in assessing how an animal will cope with a new experience or novel stimuli [3,4]. Horses constantly modify their behavior as a result of experience; this involves the creation of an association between events or stimuli [5,6].

During stressful situations such as exercise or transportation, activation of the HPA axis results in an increase of ACTH and cortisol levels. Although numerous studies have been carried out on HPA activity in horses under different exercise conditions [7-9], there is a need to assess the effect of stress induced by transport on the hormonal responses of horses.

Few of these studies were of animals being transported to slaughter under commercial conditions, with a significant rise in the concentrations of blood constituents including cortisol values, during transport, and before stunning [10,11]. Several other studies were about horses transported for different reasons such as competition, sale, breeding, and leisure activities and maintained under appropriate husbandry conditions. It is generally accepted that horses can become stressed when they travel alone, and the position facing rear to the direction of travel may be less stressful, according to different transport’s phases [12-14].

Most of this work has been concerned with the physiological response in horses following long-term transport and showing that serum cortisol concentrations after short transport were considerably higher than values registered at rest and after long distances [15], both in sport horses [16,17] and breeding stallions [18], with the highest cortisol values in calm than nervous travelled horses. Recent evidence would suggest that the comparative endocrinological responses of Equidae showed the lower HPA axis response of horses than donkey after short transportation [19] and the higher ACTH and cortisol values after conventional than simulated transportations [20].

The goal of this research has been to evaluate the HPA axis response to stress induced by transport, taking into consideration both previous transport experience and quality of the handling to which the horses were subjected. The hypothesis presented is that the quality of handling has an effect on the stress adaptation ability and on the related HPA axis response, independently of previous frequent or limited transportation experience. The study has been conducted in field conditions, so as to gain information on the real management conditions of the horses under observation.

## Materials and Methods

### Ethical approval

All methods and procedures used in this experiment were reviewed and approved by the Messina University Institutional Board for the Care and Use of Animals and were in compliance with the guidelines of the Italian Minister of Health for the care and use of animals (D.L.27/171992 n. 116) and EU (Directive 86/609/CEE) and with the regulation (EC) 1/2005 on the protection of animals during transport and related operations.

### Animals

A total of 56 clinically healthy horses, 4.4±2.5 years old, Italian saddle (Sanfratellano) and Hungarian (Gidran) breeds, 40 geldings and 16 mares, and weighing 480±60 kg were available in this study. 12 of these horses (6 Sanfratellano and 6 Gidran) were used as a control group for baseline hormonal values.

With respect to the handling quality, three categories have been determined: Good, bad, and minimal handling conditions. Horses used to physical human contact are placed in the first category. Horses which have been subjected to negative human behavior (such as slapping, hitting, and shouting) have been placed in the second category. Horses with minimal experience of human contact have been placed in the third category. The quality of human-horse relationship has been evaluated on the basis of the description of the management to which the horses were subjected.

The horses in the experimental condition (44 horses), regarding to their different housing and management conditions, were divided into four groups, Groups A, B, C, and D. With respect to the quality of previous handling experiences, Groups A and B were classified as good quality, Group C as minimal quality and while D was classified as poor quality.

Evaluation of the previous transport experience has been carried out with respect to duration and frequency of transport: Horses who have experienced long-distance road transportation (>300 km, 3-4 times during their lifetime); horses who have limited short-distance transport experience (<50 km, 1-2 times during their lifetime).

According to the previous transport, experience horses were divided as follows: Horses of Groups A and D had been experienced long-distance road transported before; horses of Groups B and C had been limited experience of transportation.

In particular, the horses of Group A, 16 Italian (Sanfratellano horses) breed, were kept by private owners on the same non-professional farm for recreational or leisure purpose. During this study, horses were submitted to transport before cross-country competition trial.

The horses of Group B, 7 Italian saddle (Sanfratellano horses) breed, were being farmed for use in trekking and riding school. During this study, they were transported for commercial markets.

The horses of Group C, 8 Italian saddle (Sanfratellano horses), breed and kept on the same livestock farm for labor production, were destined to slaughter. They had not been handled regularly since foal age and were not well accustomed to humans. During this study, they were transported to slaughter facility.

The horses of Group D, 13 meat horses (Gidran breeds), were imported from East Europe and were destined to slaughter. To improve their nutrition conditions, they were stabled in a holding place for 4 weeks before being slaughtered. Horses management was limited to food distribution and control of nutrition conditions. During this study, they were transported to slaughter facility.

About 20 horses of the control group (6 Sanfratellano and 6 Gidran) had grown up under traditional farm conditions and had been daily handled for farm routine procedures. They were kept on the same livestock farm for labor production.

All horses in the study had no history of medical problems in the preceding 2 weeks, had not received any pharmacological treatment for 2 weeks before the study and were healthy (based on physical examination).

All horses were submitted to short road transport of 30 km. The same staff handled the horses during management conditions and transport procedures. The horses were examined twice: First in their environment of home stud farms (T0), and then after transport to the new stable, at the end of each trip and unloading (T1). Control sampling was done at the same time (T0) of the day as transport.

The journeys were carried out in September on 4 different days: One trip/day for every group. All journeys were performed in the morning, between 8:30 am and 9:30 am, to avoid the diurnal rhythm of cortisol release. The duration of every trip was approximately of 45 min.

The vehicle used was a commercial eight-horse vehicle (Iveco, Turbo Zeta). Each animal was loaded into the truck without the use of force, and the loading did not last more than 5 min per horses of Groups A and B, and about 10 min per horses of Groups C and D. They stood perpendicularly to the vehicle axis, and each animal was faced alternatively in the truck. Transport started immediately after loading on the motorway with little traffic and followed the national roads on each trip.

Temperature (T°C) and relative humidity (%) inside the vehicle were continually monitored with hygrothermograph ST-50 (Model Sekonic Corporation), placed near the center of the vehicle: At start and after different trips ranged from 19.5°C to 24°C and from 62.4% to 81%, respectively.

### Blood sampling

Blood samples were collected by jugular venipuncture and were taken at the same day time (08.00 AM), immediately before loading (baseline values: T0) and after transport and unloading, at their arrival (post-transport values: T1).

Serum ACTH concentrations were analyzed in duplicate using a commercially available radioimmunoassay kit (ELSA-ACTH, CIS-Bio International, Gif-sur-Yvette, France) suitable for equine use [20]. The hormone assay used has a range for the amount of ACTH detected of 0-440 pmol/L. The intra- and inter-assay coefficients of variations (CVs) were 6% and 15%, respectively.

Serum cortisol concentrations were analyzed in duplicates using a competitive enzyme assays (Enzyme Immunoassays, Roche Diagnostics GmbH, Mannheim, Germany). Serum total cortisol concentrations were analyzed in duplicate through a competitive enzyme assay (Boehringer Mannheim Immunodiagnostic). The assay sensitivity was 5 ng/ml. The intra- and inter-assay CVs were 5.5% and 6.8%, respectively.

### Statistical analysis

Data are presented as mean±standard deviation (SD). To determine whether transport stress and previous experience had any effect a one-way analysis of variance for repeated measures (1 way RM-ANOVA) was applied. Significant differences between baseline and post-transport values, as well between baseline values of experimental and control group, were established using a Student’s paired t-test. To compare baseline values and post-transport values of different groups two-way repeated measures analysis of variance (two-way RM-ANOVA). Further changes due to the sex, breed and age were assessed by Student’s unpaired t-test. The level of significance was set at p<0.05. All calculations were performed using the PRISM package (GraphPad Software Inc., San Diego, CA).

## Results

**Figure-1:**
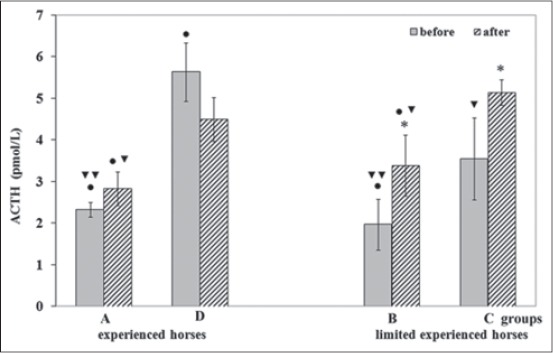
Circulating cortisol concentrations (mean ± standard deviation) of horses before and after road transport. Label x: Asterisk indicates significant (*p<0.001) differences versus before. Symbols indicate significant (•p<0.001) differences versus Group C and (^▾^p<0.01; ^▾▾^p<0.001) versus Group D.

Data obtained was presented in Figure-1. ACTH values of horses ranged between 1.97 and 5.63 pmol/L in baseline conditions and between 2.86 and 5.15 pmol/L in post-transport conditions, respectively. 1 way RM-ANOVA showed significant effects of transport on ACTH changes only in Groups B (F=23.67; p<0.01) and C (F=35.63; p<0.01).

The comparison among baseline values showed lower ACTH values in Groups A and B (p<0.01) than Groups C and D (p<0.001); the comparison among post-transport values showed lower post-transport ACTH values in Groups A and B (p<0.01) than Groups C and D (p<0.01).

The effect of horses’ previous transport experience on baseline and post-transport ACTH values was also evaluated. Baseline ACTH values of experienced horses ranged between 2.37 (Group A) and 5.63 pmol/L (Group D), and post-transport values ranged between 2.86 and 4.50 pmol/L.

Conversely, baseline ACTH values of horses’ limited experience ranged between 1.97 (Group B) and 3.55 pmol/L (Group C), and post-transport values ranged between 3.38 and 5.15 pmol/L.

Baseline ACTH values of horses of control group ranged between 2.20 (Sanfratellano breed) and 2.83 pmol/L (Gidran breed).

The comparison among baseline ACTH values of the control group and the experimental groups showed significant lower ACTH values only in the Gibran breed control horses (p<0.001) than values of horses’ Group D.

**Figure-2:**
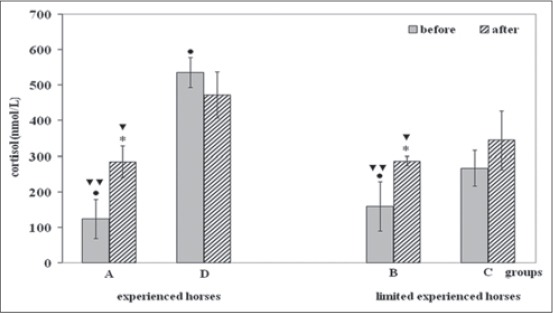
Circulating adrenocorticotropic hormone concentrations (mean±standard deviation) of horses before and after road transport. Label x: Asterisk indicates significant (*p<0.001) differences versus before, Symbols indicate significant (•p<0.001) differences versus Group C and (^▾^p<0.01; ^▾▾^p<0.001) versus Group D.

Data obtained was presented in Figure-2. Cortisol values of horses ranged between 123.22 and 529.56 nmol/L in baseline conditions and between 283.75 and 477.19 nmol/L in post-transport conditions, respectively. One-way RM-ANOVA showed significant effects of transport on cortisol changes only in Groups A (F=19.16; p<0.01) and B (F=32.78; p<0.01).

The comparison among baseline values showed lower cortisol values in Groups A and B (p<0.001) than Groups C and D; the comparison among post-transport values showed lower post-transport cortisol values in Groups A, B, and C (p<0.01) than Group D.

The effect of horses’ previous transport experience on the baseline and post-transport cortisol values was also evaluated. The baseline cortisol values of experienced horses ranged between 123.22 (Group A) and 535.34 nmol/L (Group D) and post-transport values ranged between 283.75 and 471.94 nmol/L.

Conversely, baseline cortisol values of horses’ not experience ranged between 266.14 (Group C) and 158.72 nmol/L (Group B), and post-transport values ranged between 344.50 and 286.45 nmol/L.

Baseline cortisol values of horses of control group ranged between 193.67 (Sanfratellano breed) and 203.67 nmol/L (Gidran breed).

The comparison between baseline cortisol values of the control group and the experimental groups showed significant lower cortisol values only in the Gidran breed control horses (p<0.001) than values of horses’ Group D.

There were no significant differences between the concentrations of ACTH and cortisol in all groups of experimental horses of different ages and sex, though horses ranging 4.4±2.5 years old, and mares and geldings were present in all four groups.

## Discussion

This study has been focused on the need to acquire data on HPA axis stress response to the transport in horses by monitoring circulating ACTH and cortisol concentrations compared to previous transportation and handling experiences.

The study hypothesis is that the quality of handling to which horses were subjected could affect the stress response produced by transportation more than the quality of previous transportation experiences.

To this end, we investigated the effects of different types of human handling imposed on young horses when they were kept in their stabled conditions and before short-transport road.

The results have shown an effect of different qualities of handling on cortisol basal levels, and consequently on post-transportation levels. In fact, horses with an experience of good handling have shown ACTH and cortisol basal levels lower than those of horses kept in bad or minimal handling conditions.

Furthermore, horses kept in bad or minimal handling conditions have shown ACTH and cortisol basal values higher than those of control group horses of the same breed.

Such higher levels in the minimal handling group seem to indicate a condition of perceived stress, perhaps due to the relative novelty of being handled and confined.

These results are in accordance with those reported by Petherick *et al*. who found that minimal handling/yarding appeared to cause the cattle to experience stress, while good treatment reduced stress and the poor treatment negatively impacted on live weight gain [21].

This finding probably suggests that transport-induced stress response in horses, subjected to different housing conditions, may in part be related to the quality of human–animal relationship, as shown in a previous study [22], according to which the quality of human–animal interaction in farm animals might limit the animal productivity and welfare.

Good handling conditions probably reduce the post-transportation stress perception too, reducing the stress condition caused by the novelty and uncontrollability of waiting times before transportation.

Another especially critical element is the timing, as the hormonal activity is elevated at stressor onset but reduces as time passes.

The previous studies have shown that a transport-induced stress response in horses decreased with repeated transportations, indicating that animals got used to the situation, but an increased cortisol secretion remained detectable [23].

Thus, both the quality of the experience undergone and the frequency with which it has been experienced appear to affect the capacity of stress response.

No data related to horse’s behavior during loading and unloading procedures have been directly collected in this study. Times of loading and unloading have been recorded as an indirect indication of horses behavior. Longer times have been recorded for horses from C and D groups, probably as a consequence of a greater discomfort perceived by those horses in the given conditions.

It is of particular interest the response of the transported horses, in which the familiarity with transportation has shown a diversified effect with respect to the quality of the experience undergone. Thereby, the transportation experience undergone by Group D horses has surely induced a massive HPA axis response related to the stress experienced.

Group D subjects have been sheltered for a 4 weeks period, to improve their nutrition conditions. During sheltering, the original group composition has been maintained, even when heterogeneous in sex or age.

The potential social stress could have affected the adrenergic response and modified the total and free cortisol concentrations; however, as shown in the previous studies [24], the social stress effect influences the cortisol-binding proteins concentration only in the first 2 weeks of stress exposure: The concentration then recovers its previous level in the subsequent weeks. The decrease in the post-transportation cortisol values in Group D horses could indicate a potential adrenal exhaustion, but it is more likely the effect of the ACTH-negative feedback, due to very high basal cortisol levels.

Only Groups A and B horses have shown an adequate capacity of stress response to transportation: They had transportation experiences of varied frequency but of good quality.

Accordingly, it is noted that experienced horses of Groups A and B did not modify the stress impact: Horses’ age, breed, sex and previous experience did not appear to influence it, as reported in the previous study [17,18,25].

## Conclusion

Transport produced considerable stress and its magnitude could depend on several endogenous and exogenous factors as well as the quality of horses’ previous transport and handling experience. In conclusion, the transport procedure seems to be of less importance to welfare conditions than previous human contacts during rearing. Nevertheless, transport stress responses are not linear but are the results of a physiological complex interaction of several systems. Therefore, adequate handling and management facilities, which take into account the horses’ well-being, health and welfare, could be considered to minimize stress during transport.

## Authors’ Contributions

All authors have made substantial contributions to each step of experimental procedure and manuscript preparation. In particular, the idea for the paper was conceived by EF and AF. The experiments were performed by PM and CC. The data were analyzed by PM and CC. The paper was written by EF and AF.
